# Giant Lingual Teratoma with Cleft Palate in Male New-Born

**DOI:** 10.21699/ajcr.v7i5.474

**Published:** 2016-11-01

**Authors:** J D Rawat, Piyush Kumar, Sudhir Singh, Hemlata

**Affiliations:** 1 Department of Pediatric Surgery, King George’s Medical University, Lucknow, India; 2 Department of Anesthesia, King George’s Medical University, Lucknow, India

**Dear Sir,**

Teratoma of oropharyngeal region, also known as an epignathus, is found in approximately 1:35,000–1:200,000 live births with female predominance.[1] Congenital malformations such as cleft palate, bifid tongue, dorso-nasal fistula and nasal dermoid cyst may be associated with teratoma of oropharyngeal region in 6 % cases.[2] Management of these cases are challenging with respect to handling of airway at birth and during surgery (especially with giant teratoma).

A full-term male new-born presented with a large mass arising from the oral cavity. Mass was not recognised in antenatal ultrasonography and there was no polyhydramnios. On examination, there was no respiratory distress. Patient was in stable condition. Mass was arising from the left lateral part of the tongue with little extension toward the pharynx along with cleft palate (Fig.1). Mass was variegated in appearance with some solid and cystic areas and few ill formed facial structures. CT scan revealed no extension in pharynx and there was no major vessel encasement. During Surgery, intubation was not difficult as mass was attached to the tongue. With slight traction on mass, epiglottis and vocal folds were easily visible on laryngoscopy. Complete surgical excision with tongue reconstruction was done. Tongue was held forward with stay sutures to prevent its fall for 48 hours by tacking it to sternum. Nasogastric tube feeding was started on following day and gradually shifted to spoon feeding by day-5. Patient discharged on day-7 with advice of physiotherapy of jaw. At follow up; tongue shape was maintained without any deviation. Tongue movement was good. Histopathology of excised mass revealed it mature teratoma. Patient is on follow up for cleft palate surgery.

**Figure:**
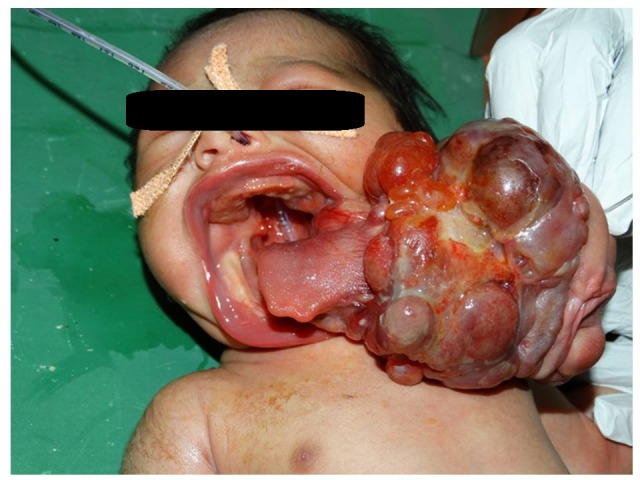
Figure 1: Giant tongue teratoma; cleft palate is well visualized.

The most common sites for head and neck teratomas are the cervical region followed by the nasopharynx, and the oropharynx. [3] Oral teratoma arising from the tongue is rare entity. More than ninety percent of head and neck teratoma usually present in neonatal and infantile period. As neonates are obligate nasal breathers, nasopharyngeal teratoma presents with respiratory distress and dysphagia and it could be lethal.[4] Oropharyngeal teratoma usually presents as difficulty in mouth closure and feeding.[5] There is no difficulty in breathing and swallowing in anterior oropharyngeal teratoma. Cases of large oral teratoma in which fetal swallowing hampered are detected antenatally due to development of polyhydramnios. There was no polyhydramnios in our case. Teratoma arising from posterior pharyngeal wall and hard palate, intracranial extension and infiltration to adjacent structure should be ruled out by CT scan. Giant teratoma of oropharynx is usually associated with cleft palate as teratoma hampers in palatal closure during in-utero development.

At birth, management of airway is key step. Presence of expert anesthetist is must to manage airway. Complete excision with preservation of adjacent structures should be the surgical goal. During excision especially with large size of tumor, injury to vital structures like lingual vessels and hypoglossal nerve are may occur.[4,5]. In our case; in spite of giant size, the tumor was excised without any deformity.

## Footnotes

**Source of Support:** Nil

**Conflict of Interest:** None declared

